# Discovery of orbital ordering in Bi_2_Sr_2_CaCu_2_O_8+*x*_

**DOI:** 10.1038/s41563-024-01817-z

**Published:** 2024-03-04

**Authors:** Shuqiu Wang, Niall Kennedy, Kazuhiro Fujita, Shin-ichi Uchida, Hiroshi Eisaki, Peter D. Johnson, J. C. Séamus Davis, Shane M. O’Mahony

**Affiliations:** 1 Clarendon Laboratory, University of Oxford, Oxford, UK; 2 Department of Physics, Cornell University, Ithaca, NY USA; 3 H. H. Wills Physics Laboratory, University of Bristol, Bristol, UK; 4 School of Physics, University College Cork, Cork, Ireland; 5 Condensed Matter Physics and Materials Science Department, Brookhaven National Laboratory, Upton, NY USA; 6 Department of Physics, University of Tokyo, Bunkyo, Japan; 7 National Institute of Advanced Industrial Science and Technology (AIST), Tsukuba, Japan; 8 Max Planck Institute for Chemical Physics of Solids, Dresden, Germany

**Keywords:** Superconducting properties and materials, Electronic properties and materials, Condensed-matter physics

## Abstract

The primordial ingredient of cuprate superconductivity is the CuO_2_ unit cell. Theories usually concentrate on the intra-atom Coulombic interactions dominating the 3*d*^9^ and 3*d*^10^ configurations of each copper ion. However, if Coulombic interactions also occur between electrons of the 2*p*^6^ orbitals of each planar oxygen atom, spontaneous orbital ordering may split their energy levels. This long-predicted intra-unit-cell symmetry breaking should generate an orbitally ordered phase, for which the charge transfer energy *ε* separating the 2*p*^6^ and 3*d*^10^ orbitals is distinct for the two oxygen atoms. Here we introduce sublattice-resolved *ε*(**r**) imaging to CuO_2_ studies and discover intra-unit-cell rotational symmetry breaking of *ε*(**r**). Spatially, this state is arranged in disordered Ising domains of orthogonally oriented orbital order bounded by dopant ions, and within whose domain walls low-energy electronic quadrupolar two-level systems occur. Overall, these data reveal a *Q* = 0 orbitally ordered state that splits the oxygen energy levels by ~50 meV, in underdoped CuO_2_.

## Abstract (web summary)

An orbitally ordered state in Bi_2_Sr_2_CaCu_2_O_8+*x*_ is revealed, which splits the energy levels of oxygen orbitals by ~50 meV.

## Main

Unforeseen and unexplained among the quantum matter states of hole-doped CuO_2_ is an electronic nematic phase^1^. Analyses of the three-orbital model^2–9^ for the CuO_2_ charge transfer insulator have long predicted a candidate mechanism for the nematic state involving energy splitting between the two planar oxygen 2*p*^6^ orbitals within each unit cell. Although never observed, such effects should be experimentally identifiable as a difference in charge transfer energy *ε*(**r**) separating each oxygen 2*p*^6^ orbital from the relevant copper 3*d*^10^ orbital configuration. Moreover, if extant, such ordering of the oxygen 2*p*^6^ orbitals may be juxtaposed with iron-based high-temperature superconductors where intra-unit-cell rotational symmetry breaking between the iron *d*_*zx*_ and *d*_*zy*_ orbitals^10–12^ is pivotal^13,14^.

Soon after the discovery of Fe-based high-temperature superconductivity, the profound significance of ordering in the iron *d*_*zx*_ and *d*_*zy*_ orbitals was identified^10–12^, along with the subsequent realization that such orbital ordering (lifting energy degeneracy of *d*_*zx*_ and *d*_*zy*_ orbitals) was a fundamentally important phenomenon^13,14^. In these materials, such orbital ordering is pivotal for the tetragonal-to-orthorhombic phase transition into an electronic nematic phase^1,13^. This lifting of *d*_*zx*_:*d*_*zy*_ energy degeneracy certainly has profound global effects^13,14^: sequentially with falling temperature, the orbital ordering renders the electronic structure strongly nematic along with a strongly anisotropic antiferromagnetic state; its spectrum of magnetic fluctuations exhibits equivalently reduced symmetry^13^; finally, a strongly anisotropic^14^ and even orbital-selective^15^ form of high-temperature superconductivity emerges. Hence, intra-unit-cell orbital ordering produces powerful, wide-ranging effects on the electronic structure, quantum magnetism, high-temperature superconductivity and on the global phase diagram of the Fe-based high-temperature superconductive materials^13,14,16,17^. However, although long anticipated^2–9^, analogous intra-unit-cell orbital ordering for Cu-based high-temperature superconductive materials has never been observed.

## Charge transfer superexchange

**Fig. 1:**
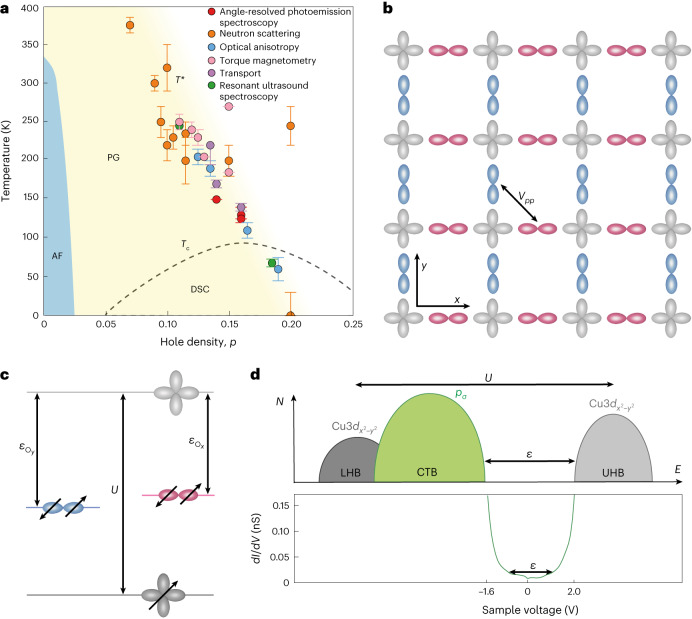
Concept of CuO_2_ intra-unit-cell orbital order. **a**, Signatures of nematic phase appearing at the cuprate pseudogap (PG) temperature T*(*p*). Each data point is the average of multiple measurements from each technique (Methods). AF represents the antiferromagnetic state and DSC represents d-wave superconductivity. The error bars are accordingly derived from these estimates. **b**, Schematic of the relevant orbitals in the CuO_2_ plane depicting the crucial inter-oxygen-orbital Coulomb interaction *V*_*pp*_. **c**, Degeneracy of the Cu $3d_{x^2-y^2}$ orbital (grey) is lifted by the Coulomb energy *U*. The *p*_*x*_ orbital of the oxygen along the *x* axis of the Cu atom (red) is separated from the upper Cu band by the charge transfer energy *ε*_*x*_. At the oxygen site along the *y* axis from the Cu atom (blue), the *p*_*y*_ orbital is separated from the upper Cu band by the charge transfer energy *ε*_*y*_. **d**, Schematic of the density of electronic states (top) where the Coulomb energy *U* and charge transfer energy *ε* are indicated. Typical measured differential conductance *g*(*V*) spectrum (bottom) where the top of the lower band and the bottom of the upper band are visualized, their separation being a direct measure of charge transfer energy *ε* (ref. ^25^). CTB represents the charge transfer band, LHB represents the lower Hubbard band and UHB represents the upper Hubbard band. Source data

In these materials, planar Cu^++^ ions are in the 3*d*^9^ configuration with a singly occupied $d_{x^2-y^2}$ orbital, whereas the planar O^−−^ ions have filled 2*p*^6^ orbitals. The Cu 3*d*^10^ configuration, which is energetically disfavoured by the Coulomb energy *U* required to doubly occupy each $d_{x^2-y^2}$ orbital, is separated from the intervening oxygen 2*p*^6^ energy levels by the charge transfer energy *ε* (Fig. 1d). Although at half-filling, the *d* electrons are fully localized in a charge transfer insulator state, the introduction of holes into the oxygen 2*p*^6^ orbitals radically alters the situation, necessitating a three-orbital Hamiltonian^18,19^ description:

1 $$H=\sum _{i\alpha j\beta ,\sigma }{t}_{{ij}}^{\alpha \beta }{c}_{i\sigma }^{\dagger \alpha }{c}_{j\sigma }^{\;\beta }+\sum _{i\sigma ,\alpha }{\varepsilon }_{\alpha }{n}_{i\sigma }^{\alpha }+U\sum _{i}{n}_{i\uparrow }^{d}{n}_{i\downarrow }^{d}.$$

**Extended Data Fig. 1:**
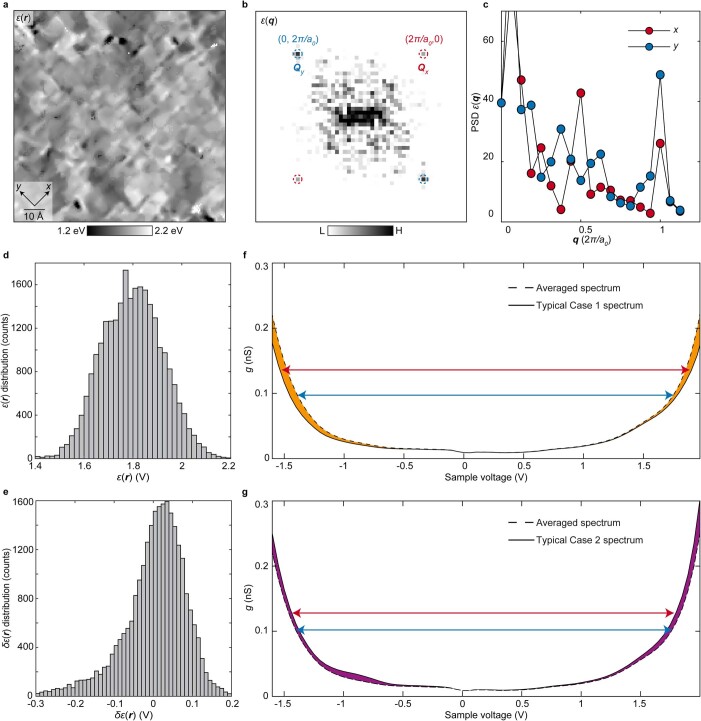
Validity of the algorithm to measure the charge transfer energy variations *δε*(*r*). (**a**) Image of the charge transfer energy ${\varepsilon }\left({\boldsymbol{r}}\right)$ calculated from Eq. 4. The supermodulation (~150 meV) wavevector ${{\boldsymbol{Q}}}_{{\rm{SM}}}$ is masked in (a) such that the intra-unit-cell features (~50 meV) are revealed. (**b**) Fourier transform of ${\varepsilon }\left({\boldsymbol{r}}\right)$. (**c**). Linecut from (0,0) to the Bragg peaks in ${\varepsilon }\left({\boldsymbol{r}}\right)$, where the ratio of the Bragg peaks intensity $\frac{{{\boldsymbol{Q}}}_{y}}{{{\boldsymbol{Q}}}_{x}}=1.9$ indicates IUC symmetry breaking in charge transfer energy ${\varepsilon }\left({\boldsymbol{r}}\right)$. (**d**) Statistical distribution of ${\varepsilon }\left({\boldsymbol{r}}\right)$. (**e**) Statistical distribution of $\delta {\varepsilon }\left({\boldsymbol{r}}\right)$. The narrower distribution in $\delta {\varepsilon }\left({\boldsymbol{r}}\right)$ demonstrates an improvement in signal-to-noise ratio over the ${\varepsilon }\left({\boldsymbol{r}}\right)$ algorithm. The histogram of $\delta {\varepsilon }\left({\boldsymbol{r}}\right)$ has a narrower σ than the histogram of ${\varepsilon }\left({\boldsymbol{r}}\right)$, demonstrating the signal-to-noise ratio is higher in the algorithm used here to measure the variations in charge transfer energy $\delta {\varepsilon }\left({\boldsymbol{r}}\right)$. Thus this $\delta {\varepsilon }\left({\boldsymbol{r}}\right)$ algorithm with higher SNR was used throughout this paper. (**f-g**) Typical examples of the splitting of the charge transfer energy. (**f**) shows a case when the energy variation is positive, $\delta {\varepsilon } > 0$. (**g**) shows a case when the energy variation is negative, $\delta {\varepsilon } < 0$.

Here *α* and *β* label any of the three orbitals, ${t}_{{ij}}^{\alpha \beta }/\hslash$ are transition rates for electrons between orbitals *α* and *β* at sites *i* and *j*, *ε*_*α*_ are the orbital energies and ${n}_{i\uparrow }^{d},{n}_{i\downarrow }^{d}$ are the *d*-orbital occupancies. High-temperature superconductivity is believed to emerge within this model driven by the charge transfer superexchange pairing interactions between the Cu *d* electrons (Methods and Extended Data Fig. 1). In theory^1^, however, many other ordered phases could emerge on the hole doping of CuO_2_, with a planar oxygen orbitally ordered phase^2–9^ due to additional inter-oxygen repulsion terms ${V}_{{pp}}{\sum }_{{ij}\sigma \sigma {\prime} }{n}_{i\sigma }^{p}{n}_{j\sigma {\prime} }^{p}$ (equation (1) being a prime example).

One enigmatic phase that does emerge is the pseudogap regime. Its essential phenomenology^20,21^ is that for *T* < T*(*p*) and *p* < p* ≈ 0.19 (Fig. 1a, yellow shading), the Fermi surface becomes partially gapped, thereby diminishing the spectrum of electronic states, magnetic susceptibility, electronic specific heat, *c*-axis electrical conductivity and spatially averaged density of electronic states. Strikingly, however, in this same region of the phase diagram, there is pervasive evidence for a nematic state in which the electronic structure breaks 90°-rotational (*C*_4_) symmetry at wavevector **Q** = 0, or equivalently within the CuO_2_ unit cell. Experimental signatures of this nematic state have been widely reported based on multiple techniques (Fig. 1a), and this nematic phenomenology is observed for *p* < p* in the Bi_2_Sr_2_CaCu_2_O_8+*x*_, Bi_2_Sr_2_CuO_6+*x*_, Bi_2–*z*_Pb_z_Sr_2–*y*_La_*y*_CuO_6+*x*_, YBa_2_Cu_3_O_7–*x*_, La_2–*x*_Ba_*x*_CuO_4_ and HgBa_2_CuO_4+*δ*_ material families, thereby strongly indicative of universality (Methods). However, no microscopic mechanism has yet been experimentally established for this CuO_2_ nematic phase.

## Motivation for cuprate orbital ordering

Classically, a nematic metallic state may occur due to the Pomeranchuk instability of the Fermi surface^22^. But, for cuprates, theoretical analyses using a three-orbital band structure^2–9^ predict the emergence of low-energy electronic nematicity primarily driven by high-energy orbital ordering. Specifically, when the Coulomb repulsions between electrons on nearest-neighbour O sites within the same unit cell are included in the strong-coupling limit^2^ and in self-consistent mean-field theory^3^, an orbitally ordered nematic phase is predicted. On the same microscopic basis, such CuO_2_ orbital order is also predicted using diagrammatic perturbation theory^4^, perturbative expansion^5^, functional renormalization group techniques^7^, Hartree–Fock-based models^8^ and slave-boson mean-field theory^9^. Figure 1b conceptually represents such planar oxygen orbital order, whereas Fig. 1c represents a schematic of its impact on the intra-unit-cell characteristics of charge transfer energy *ε*. Here the 2*p*^6^ orbital of the oxygen atom along the CuO_2_ *x* axis (O_*x*_ site) is separated from the upper Cu band by the charge transfer energy *ε*_*x*_, whereas the notionally equivalent oxygen orbital along the *y* axis (O_*y*_ site) exhibits a different charge transfer energy *ε*_*y*_. Our objective is then a direct search for such rotational symmetry breaking at the charge transfer energy scale by the visualization of *ε* within each CuO_2_ unit cell.

## Visualization of charge transfer energy variations

**Fig. 2:**
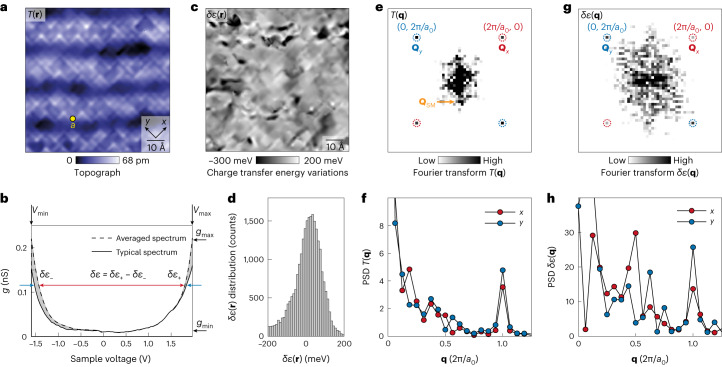
Visualizing CuO_2_ charge transfer energy variations δ*ε*(**r**). **a**, Topograph of the BiO surface of Bi_2_Sr_2_CaCu_2_O_8+*x*_ (*V*_S_ = –750 mV:*I*_S_ =25 pA). The bulk-crystal supermodulation, a quasi-periodic modulation along the (1, 1) direction, is clearly evident. It is at 45° to—and therefore is the mirror plane between—the *x* and *y* axes, as always^26^. Any distinctions between the states of oxygen orbitals along the *x* and *y* axes are not influenced by supermodulation for this symmetry reason, as empirically demonstrated in Methodsand Extended Data Fig. 9. Therefore, supermodulation has no discernable influence on the intra-unit-cell symmetry breaking of δ*ε*(**r**). **b**, High-voltage differential conductance spectra *g*(**r**, *V*) are shown as a solid black curve, whereas the spatially averaged spectrum $\overline{g\left(V\right)}$ is shown as a dashed curve. The example spectrum is measured at a location (yellow dot) in **a**. Such high junction resistances of 85 GΩ or large tip–sample distances preclude the effects of the tip–sample electric field on *g*(*V*). The separation between the lower and upper bands is clearly visible for the example spectrum (blue arrows) as well as for the average spectrum (red double-headed arrow). Setpoint *V*_S_ = –600 mV:*I*_S_ = 7 pA. **c**, Visualization of charge transfer energy variations δ*ε*(**r**) from **a**. **d**, Histogram of charge transfer energy variations δ*ε* in **c**. **e**, PSD Fourier transform *T*(**q**) of the topograph measured simultaneously as **c**. The **Q**_SM_ peaks (orange arrow) signify the supermodulation. **f**, Linecuts from **q** = (0, 0) to (1, 0)2π/*a*_0_ and from **q** = (0, 0) to (0, 1)2π/*a*_0_ in *T*(**q**). The values at **Q**_*x*_ = (1, 0)2π/*a*_0_ and **Q**_*y*_ = (0, 1)2π/*a*_0_ are indistinguishable; thus, the PSD *T*(**q**) does not break *C*_4_ symmetry at its Bragg peaks. **g**, PSD Fourier transform δ*ε*(**q**) of charge transfer energy map from **c**. The **Q**_SM_ peaks are removed in δ*ε*(**q**). **h**, Linecuts from **q** = (0, 0) to (1, 0)2π/*a*_0_ and from **q** = (0, 0) to (0, 1)2π/*a*_0_ in δ*ε*(**q**) from **g**. The δ*ε*(**q**) breaks *C*_4_ symmetry at its Bragg peaks as the plots of δ*ε*(**q**) are distinct at **Q**_*x*_ = (1, 0)2π/*a*_0_ and **Q**_*y*_ = (0, 1)2π/*a*_0_. This is direct evidence of intra-unit-cell rotational symmetry breaking at the charge transfer energy in cuprates. Source data

**Extended Data Fig. 2:**
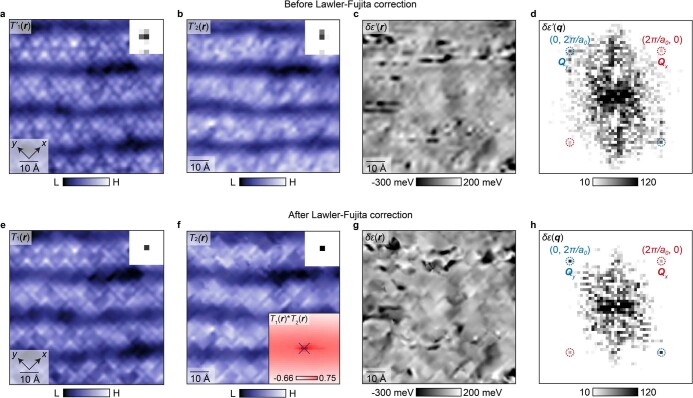
Comparison between unprocessed data and Lawler-Fujita corrected data of Bi_2_Sr_2_CaCu_2_O_8+*x*_. (**a** & **b**) Show two unprocessed low and high voltage topographs in the same field of view. The low-voltage topograph *T*'_1_(***r***) (a) has atomic resolution and it allows Bi atoms to be identified (R_N_ - 30 GΩ, V_S_ = −750 mV). The high voltage topograph *T*'_2_(***r***) in (b) was acquired simultaneously with the electronic structure $g({\boldsymbol{r}},V)$ (R_N_ - 85 GΩ, V_S_ = 600 mV). The insets show the broad Bragg peaks of its topo. (**c**) is an unprocessed map of the charge transfer energy $\delta {\varepsilon}^{\prime} \left({\boldsymbol{r}}\right)$ and (**d**) is the corresponding PSD Fourier transform. $\delta {\varepsilon}^{\prime} \left({\boldsymbol{q}}\right)$ shows anisotropy between its Bragg peaks, which are marked by dashed circles, with $\frac{{{\boldsymbol{Q}}}_{y}}{{{\boldsymbol{Q}}}_{x}}=$ 2.0. (**e** & **f**) show the same topographs after Lawler-Fujita correction. Insets show sharp Bragg peaks indicating that piezo drift has been corrected. Inset: Zoom-in image (center) of the cross-correlation between ${T}_{1}\left({\boldsymbol{r}}\right)$ and ${T}_{2}\left({\boldsymbol{r}}\right)$. The maximum (annotated by the cross) has a width of 3 pixels. The registration precision is better than 80 pm (equivalent to 1.5 pixels). (**g**) is $\delta {\varepsilon }\left({\boldsymbol{r}}\right)$ after Lawler-Fujita correction for piezo-drift and (**h**) the corresponding PSD Fourier transform. The Bragg peaks are single pixel and IUC symmetry breaking remains present at the Bragg peaks with $\frac{{{\boldsymbol{Q}}}_{y}}{{{\boldsymbol{Q}}}_{x}}=1.9.$ The disorder at ${\boldsymbol{q}}=$ 0 has been noticeably reduced.

In practice, Bi_2_Sr_2_CaCu_2_O_8+*x*_ samples with hole density *p* ≈ 0.17 and *T*_c_ = 88 K are inserted into a spectroscopic imaging scanning tunnelling microscope and cleaved in a cryogenic ultrahigh vacuum at *T* = 4.2 K. The technique of imaging charge transfer energies in cuprates has been established by several scanning tunnelling microscopy studies^23–25^ of variations in the d*I*/d*V* spectra measured over the energy range of −1.5 < *E* < 2.0 eV. However, intra-unit-cell resolution imaging of the charge transfer energies has proven to be challenging due to the high tunnelling junction resistance (and thus large tip–sample separation) required for such high-voltage imaging. Here we overcome such challenges and introduce sublattice-resolved^26–29^ imaging of *ε*(**r**) into CuO_2_ studies. A typical topographic image, *T*(**r**), of the terminal BiO surface from these studies is shown in Fig. 2a. To visualize the intra-unit-cell structure of the charge transfer energy scale, we use a recently developed technique^25^ that analyses high-voltage tunnelling conductance spectra *g*(**r**, *E*) ≡ d*I*/d*V*(**r**, *eV*) so as to yield the spatial variations in *ε*(**r**). Figure 2b shows an example of a high-voltage *g*(**r**, *V*) spectrum, measured using junction resistance *R*_N_ ≈ 85 GΩ at a specific site **r** within the CuO_2_ unit cell (Fig. 2a, yellow dot). Compared with the spatially averaged $\overline{g\left(V\right)}$ in the same field of view (FOV), we see that they have different energy separations between the lower band (filled) and upper band (empty) (Fig. 2b, red and blue arrows). Hence, by visualizing *g*(**r**, *V*) in the *V*_min_ (–1.6 V) ≤ *V* ≤ *V*_max_ (2.0 V) range at these junction resistances, one can find departures in *ε*(**r**) from the average, for every unit cell in the FOV. To do so, we first use Fourier filtering to remove the structural supermodulation with wavevector **Q**_SM_ from the measured *g*(**r**, *V*); this is necessary because supermodulation is known to modulate the charge transfer energy^25^ with an amplitude of approximately 150 meV. We then integrate each measured *g*(**r**, *V*) followed by the evaluation of ${I}_{+}({\bf{r}})={\int }_{0}^{{V}_{\max }}g({\mathbf{r}},V){\mathrm{d}}V$, the integrated density of states giving rise to the current for all positive-energy states and similarly for the negative-energy states, ${I}_{-}({\bf{r}})={\int }_{{V}_{\min }}^{0}g({\bf{r}},V\;){{\rm{d}}V}$, where $\bar{{I}_{\pm }}$ are the equivalent integrals for the spatially averaged spectrum $\overline{g\left(V\right)}$. We normalize the current by the junction resistance, which is given by 1/(*g*_max_ – *g*_min_), consequently leading to energy variations in the charge transfer band. Both filled and empty states are used in this calculation (Methods and Extended Data Fig. 1). Next, to estimate the variations in the charge transfer energy away from its mean, we use

2 $$\delta {\varepsilon }{^{\prime}}({\bf{r}})\equiv \left[\left(\bar{{I}_{+}}-{I}_{+}({\bf{r}})\right)-\left(\bar{{I}_{-}}-{I}_{-}\left({\bf{r}}\right)\right)\right]/\left({g}_{\max }-{g}_{\min }\right),$$

which averages over a wide range of tunnel conductances *g* between *g*_min_ (0.01 nS) and *g*_max_ (0.22 nS). This integration algorithm is demonstrated to provide an improved signal-to-noise ratio (Methods and Extended Data Fig. 1) in measuring the variations in charge transfer energy compared with the previous algorithm^25^. Finally, the Lawler–Fujita (LF) procedure^26^ is used to morph the simultaneously measured topograph *T*′(**r**) into a perfectly periodic tetragonal image *T*(**r**) (Fig. 2a, Methods and Extended Data Fig. 2), in which the geometry of every unit cell is equivalent; the identical transformation carried out on δ*ε*′(**r**) yields an image of charge transfer energy variations δ*ε*(**r**) that are equally unit-cell periodic^26–29^. This δ*ε*(**r**) and its power spectral density (PSD) Fourier transform δ*ε*(**q**) become the basis for studies of intra-unit-cell symmetry breaking, where Fig. 2c shows the configuration of δ*ε*(**r**) measured in the FOV shown in Fig. 2a. The distribution of the measured δ*ε* values (Fig. 2c) is shown in Fig. 2d and yields a root mean square (r.m.s.) value of δ*ε*_r.m.s._ ≈ 90 meV. As demonstrated below, this distribution is dominated by the spatial arrangements of an intra-unit-cell ordered state.

## Intra-unit-cell symmetry breaking of charge transfer energy

**Extended Data Fig. 3:**
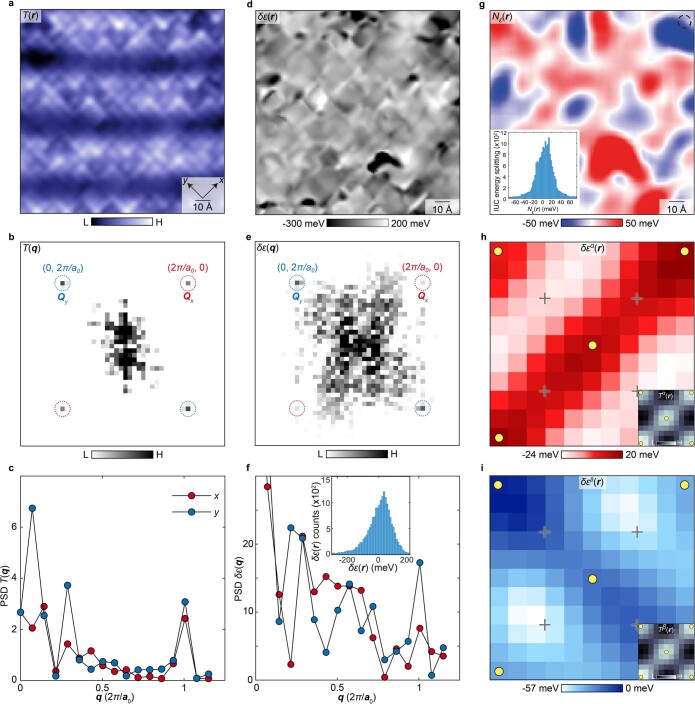
Fourier analysis of charge-transfer energy variations map *δε*(*r*) in another FOV. (**a**) Second FOV of Bi_2_Sr_2_CaCu_2_O_8+*x*_ surface topography, *T*(**r**). (**b**) *T*(**q**), the Fourier transform PSD of (a). (**c**) Linecuts of $T\left({\boldsymbol{q}}\right)$ in **Q**_*x*_ (red) and **Q**_*y*_ (blue) directions show the ratio of the transverse averaged intensity at the Bragg peaks is $\frac{{{\boldsymbol{Q}}}_{y}}{{{\boldsymbol{Q}}}_{x}}=1.3$. (**d**) Second FOV of charge transfer energy variations, *δε*(**r**). (**e**) *δε*(**q**), the Fourier transform PSD of (d). (**f**) Linecuts of $\delta {\varepsilon }({\boldsymbol{q}})$ show $\frac{{{\boldsymbol{Q}}}_{y}}{{{\boldsymbol{Q}}}_{x}}=2.3$ and ${\delta {\varepsilon }}_{{RMS}}=95$ meV. Thus the charge transfer energy variations break rotational symmetry. (**g**) Nematicity analysis of the same FOV in (**a**). Image of oxygen-site-specific nematic order parameter ${N}_{\varepsilon }\left({\boldsymbol{r}}\right)$. The area ratio ${A}_{{red}}/{A}_{{blue}}$ is 1.8. The largest domain size is ~29 nm^2^. Inset: histogram of ${N}_{\varepsilon }\left({\boldsymbol{r}}\right)$ whose RMS is ~28 meV. (**h**-**i**) Unit cell averaged structure of charge transfer energy variations $\delta {\varepsilon }({\boldsymbol{r}})$ from two Ising domains. Inset: unit cell averaged structure of $T\left({\boldsymbol{r}}\right)$ of each Ising domain. The yellow circles represent the location of the Cu sites.

**Extended Data Fig. 4:**
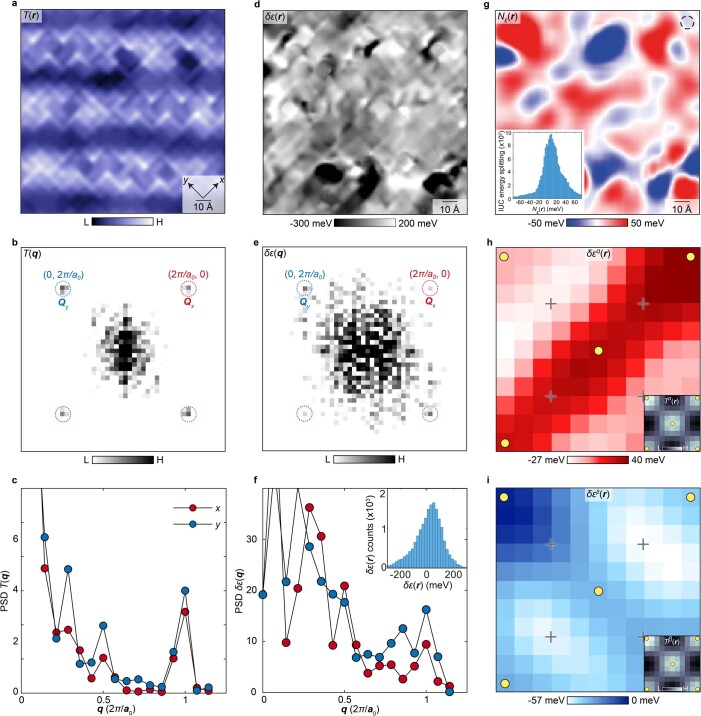
Fourier analysis of charge transfer energy variations map *δε*(*r*) in another FOV. (**a**) Third FOV of Bi_2_Sr_2_CaCu_2_O_8+*x*_ surface topography, *T*(**r**). (**b**) *T*(**q**), the Fourier transform PSD of (a). (**c**) Linecuts of $T({\boldsymbol{q}})$ show the ratio of the transverse averaged intensity at the Bragg peaks is $\frac{{{\boldsymbol{Q}}}_{y}}{{{\boldsymbol{Q}}}_{x}}=1.3$. (**d**) Third FOV of charge transfer energy variations, *δε*(**r**). (**e**) *δε*(**q**), the Fourier transform PSD of (d). (**f**) Linecuts of $\delta {\varepsilon }({\boldsymbol{q}})$ show the intensity ratio of $\frac{{{\boldsymbol{Q}}}_{y}}{{{\boldsymbol{Q}}}_{x}}=1.7$ and ${\delta {\varepsilon }}_{{RMS}}$ ${\rm{is}}$ $\sim 129$ meV. Thus the charge transfer energy variations break rotational symmetry. (**g**) Nematicity analysis of the same FOV in (**a**). Image of oxygen-site-specific nematic order parameter ${N}_{\varepsilon }\left({\boldsymbol{r}}\right)$. The area ratio ${A}_{{red}}/{A}_{{blue}}$ is 2.3. The largest domain size is ~33 nm^2^. Inset: histogram of ${N}_{\varepsilon }\left({\boldsymbol{r}}\right)$ whose RMS is ~34 meV. (**h**-**i**) Unit cell averaged structure of charge transfer energy variations $\delta {\varepsilon }({\boldsymbol{r}})$ from two Ising domains. Inset: unit cell averaged structure of $T\left({\boldsymbol{r}}\right)$ from each Ising domain. The yellow circles represent the location of the Cu sites.

**Extended Data Fig. 5:**
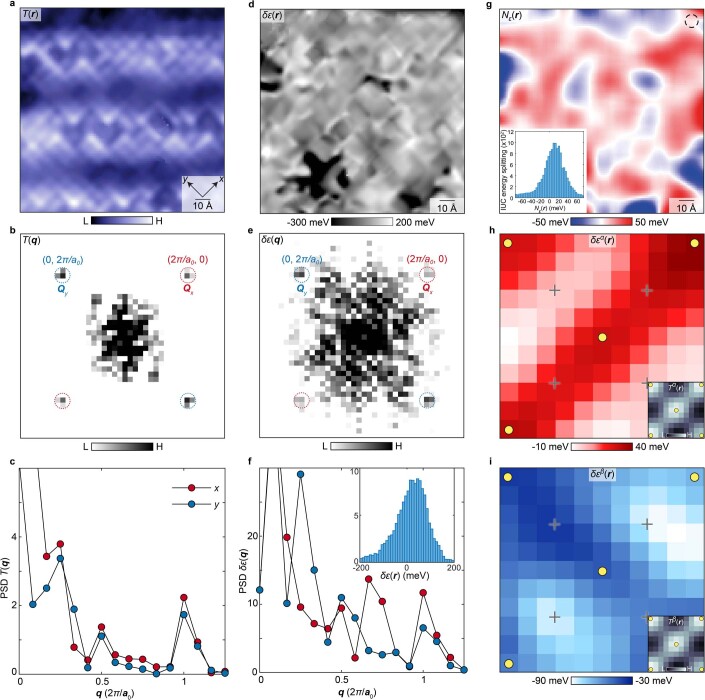
Fourier analysis of charge-transfer energy variations map *δε*(*r*) in another FOV. (**a**) Fourth FOV of Bi_2_Sr_2_CaCu_2_O_8+*x*_ surface topography, *T*(**r**). (**b**) *T*(**q**), the Fourier transform PSD of (a). (**c**) Linecuts of $T({\boldsymbol{q}})$ show the ratio of the transverse averaged intensity at the Bragg peaks is $\frac{{{\boldsymbol{Q}}}_{y}}{{{\boldsymbol{Q}}}_{x}}=1.3$. (**d**) Fourth FOV of charge transfer energy variations, *δε*(**r**). (**e**) *δε*(**q**), the Fourier transform PSD of (d). (**f**) $\delta {\varepsilon }({\boldsymbol{q}})$ shows $\frac{{{\boldsymbol{Q}}}_{y}}{{{\boldsymbol{Q}}}_{x}}=1.8$ and ${\delta {\varepsilon }}_{{RMS}}\approx 105$ meV. Thus the charge transfer energy variations break rotational symmetry. (**g**) Nematicity analysis of the same FOV in (**a**). Image of oxygen-site-specific nematic order parameter ${N}_{\varepsilon }\left({\boldsymbol{r}}\right)$. The area ratio ${A}_{{red}}/{A}_{{blue}}$ is 2.2. The largest domain size is ~23 nm^2^. Inset: histogram of ${N}_{\varepsilon }\left({\boldsymbol{r}}\right)$ whose RMS is ~26 meV. (**h**-**i**) Unit cell averaged structure of charge transfer energy variations $\delta {\varepsilon }({\boldsymbol{r}})$. Inset: unit cell averaged structure of $T\left({\boldsymbol{r}}\right)$ from the corresponding Ising domain. The yellow circles represent the location of the Cu sites.

**Extended Data Fig. 6:**
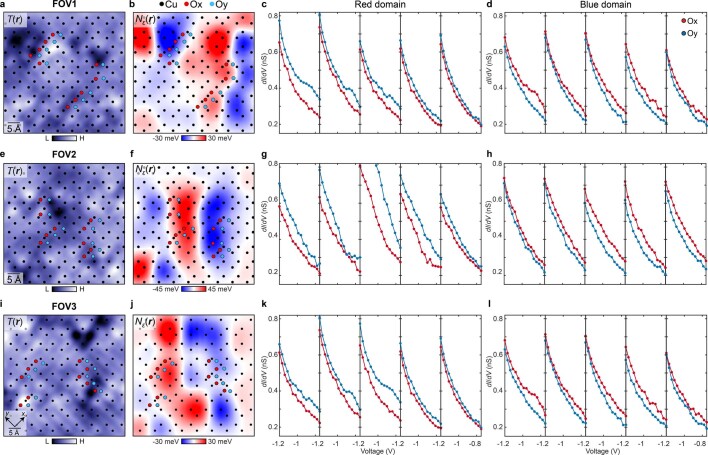
Intra-unit-cell splitting of unprocessed differential conductance spectra. (**a, e, i**) First column: Topograph of the FOV where the spectra are measured. (**b, f, j**) Second column: Image of nematic intra-unit-cell order parameter ${N}_{\varepsilon }\left({\boldsymbol{r}}\right)$ sampled on oxygen sites. Two Ising domains are labelled. (**c, g, k**) Third column: 10 dI/dV point spectra sampled on the oxygen sites from Domain 1 in (b). Clearly these point spectra show the Ox spectra are shifted by -50 meV ~ -30 meV with respect to their intra-unit-cell Oy spectra. The locations of the Ox and Oy sites are shown as circles in (a-b). (**d, h, l**) Fourth column: 10 point spectra sampled on the oxygen sites from Domain 2 reveal that the Oy spectra are shifted by -50 meV ~ -30 meV with respect to the Ox spectra from the same unit cell. The locations of the unit cell and oxygen sites are shown in circles in (a-b). The dI/dV splitting to opposite directions in the two different domains, proves charge transfer energy splitting occurs in unprocessed differential conductance spectra.

Next, we explore the symmetry of intra-unit-cell structure of δ*ε*(**r**) by studying the values of δ*ε*(**q**) measured at the two symmetry-inequivalent Bragg peaks ${{\bf{Q}}}_{{\bf{x}}}^{{\rm{B}}}=2\uppi /{a}_{0}(\mathrm{1,0})$ and ${{\bf{Q}}}_{{\bf{y}}}^{{\rm{B}}}{\boldsymbol{=}}2\uppi /{a}_{0}(\mathrm{0,1})$ of the CuO_2_ plane^26,27^. Figure 2e shows the measured PSD Fourier transform of the simultaneous topograph *T*(**q**) and Fig. 2f shows these data plotted along the two orthogonal axes. The magnitudes of the two Bragg peaks are indistinguishable (*T*$\left({{\bf{Q}}}_{{\bf{x}}}^{{\rm{B}}}\right){\boldsymbol{\approx }}T\left({{\bf{Q}}}_{{\bf{y}}}^{{\rm{B}}}\right)$) within the error bars, meaning that neither the crystal nor the scanning tunnelling microscope tip breaks the intra-unit-cell rotational symmetry. In contrast, Fig. 2g shows the measured magnitude of δ*ε*(**q**), whereas Fig. 2h shows these data plotted along the two orthogonal axes. Now, the magnitudes of the two Bragg peaks in δ*ε*(**q**) are different (Methods and Extended Data Figs. 3–5 show a repeatable observation), revealing the existence of **Q** = 0 rotational symmetry breaking of the electronic structure at the charge transfer energy range in Bi_2_Sr_2_CaCu_2_O_8+*x*_. To double check, by intra-unit-cell imaging in real space, we also directly demonstrate that this rotational symmetry breaking is a valid empirical property at the charge transfer energy scale of all the unprocessed high-voltage sublattice-resolved d*I*/d*V*(**r**, *eV*) data (Methods and Extended Data Fig. 6).

## Ising domains of orbital ordering

**Fig. 3:**
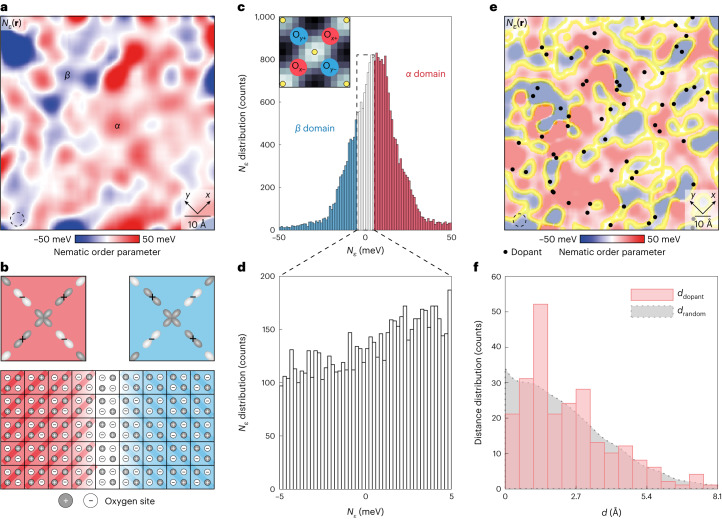
Intra-unit-cell oxygen-specific characteristics of charge transfer energy. **a**, Image of nematic intra-unit-cell order parameter *N*_*ε*_(**R**_**i**__**j**_) sampled on oxygen sites. Here ${\delta {\varepsilon }}_{{{{\rm{O}}_x}}{{({\rm{O}}_y)}}}$ represent the four oxygen sites of each unit cell (inset in **c**) from the FOV shown in Fig. 2a. The continuous *N*_*ε*_(**r**) image is achieved by the Gaussian smoothing of *N*_*ε*_(**R**_**i**__**j**_) with a radius of 3.2 Å (dashed circle). The powerful breaking of intra-unit-cell *C*_4_ symmetry is clearly observed in δ*ε*(**r**), whereas the predominant disorder is revealed as Ising domains of opposite sign *N*_*ε*_. **b**, Schematic of the charge-quadrupole two-level systems within the Ising-domain walls. The top panel shows the schematic of the charge distribution in the four intra-unit-cell oxygen sites consequent to the intra-unit-cell charge transfer symmetry breaking. The bottom panel shows a schematic of two orbitally ordered domains consisting of orthogonally oriented charge quadrupoles, and a domain wall within which the lower-energy barrier to fluctuations in the charge quadrupolar orientation can be exceeded at finite temperatures. The darker circles indicate oxygen sites with higher charge transfer energy *ε*, whereas lighter circles represent those with lower *ε*. **c**, Histogram of *N*_*ε*_(**r**) in **a** with an r.m.s. value of 25 meV. This histogram represents a combination of two populations of ordered Ising nematic domains plus all the randomized values of δ*ε*(**r**) within the intervening domain walls. The inset shows the unit cell sampled on four oxygen sites (red along the *x* axis and blue along the *y* axis) for calculating the nematic order parameter, with Cu sites marked by yellow circles. **d**, Histogram *N*_*ε*_ from all the non-ordered regions of **a**. Such regions are identified as the white domain walls in **a**, where |*N*_*ε*_| < 5 meV. The thermal energy barrier of 5 meV is equivalent to approximately 60 K. **e**, Location of oxygen dopant ions (black dots) measured simultaneously as *N*_*ε*_(**r**) (Methods and Extended Data Fig. 7). They occur proximate to the domain walls (yellow contours) where |*N*_*ε*_(**r**)| < 5 meV. **f**, Histogram of the shortest dopant ion–domain-wall distance *d*_dopant_ (pink bars). The histogram of the expected averaged distance *d*_random_ between the simulated Poisson random point and its nearest domain-wall point (grey area), uncorrelated with *N*_*ε*_(**r**) (Methods and Extended Data Fig. 7). Source data

This motivates the development of a CuO_2_ orbital order parameter for the efficient visualization of intra-unit-cell energy-splitting variations. We specifically focus on the two planar oxygen atom sites within each CuO_2_ unit cell. First, we sample the values of δ*ε*(**r**) surrounding the oxygen atom sites along the *x* axis from Cu atom **R**_**ij**_ and label them ${\delta {\varepsilon }}_{{{\rm{O}}_x}}\left({{\bf{R}}}_{{\bf{ij}}}{\bf{-}}a\hat{{\bf{x}}}/2\right)$: ${\delta {\varepsilon }}_{{{\rm{O}}_x}}\left({{\bf{R}}}_{{\bf{ij}}}{\bf{-}}a\hat{{\bf{x}}}/2\right)$; equivalently, we sample the two nearest planar oxygen atom sites along the *y* axis from **R**_**ij**_ and label them ${\delta {\varepsilon }}_{{{\rm{O}}_y}}\left({{\bf{R}}}_{{\bf{ij}}}{\bf{-}}a\hat{{\bf{y}}}/2\right)$:${\delta {\varepsilon }}_{{{\rm{O}}_y}}\left({{\bf{R}}}_{{\bf{ij}}}{\bf{-}}a\hat{{\bf{y}}}/2\right)$ (Fig. 3c, inset), where *a* is the CuO_2_ lattice constant. Using this approach, a nematic order parameter *N*_*ε*_(**r**) may be defined^26,30^ as the difference between the average ${\delta {\varepsilon }}_{{{\rm{O}}_x}}({\bf{r}})$ and ${\delta {\varepsilon }}_{{{\rm{O}}_y}}({\bf{r}})$ inside each unit cell:

3 $${N}_{{\varepsilon }}\left({{\bf{R}}}_{{\bf{ij}}}\right)=\begin{array}{l}\frac{\left[{\delta {\varepsilon }}_{{{\rm{O}}_x}}\left({{\bf{R}}}_{{\bf{ij}}}{\bf{+}}a\hat{{\bf{x}}}/2\right)+{\delta {\varepsilon }}_{{{\rm{O}}_x}}\left({{\bf{R}}}_{{\bf{ij}}}{\bf{-}}a\hat{{\bf{x}}}/2\right)\right]}{2}-\frac{\left[{\delta {\varepsilon }}_{{{\rm{O}}_y}}\left({{\bf{R}}}_{{\bf{ij}}}{\bf{+}}a\hat{{\bf{y}}}/2\right)+{\delta {\varepsilon }}_{{{\rm{O}}_y}}\left({{\bf{R}}}_{{\bf{ij}}}{\bf{-}}a\hat{{\bf{y}}}/2\right)\right]}{2}.\end{array}$$

**Extended Data Fig. 7:**
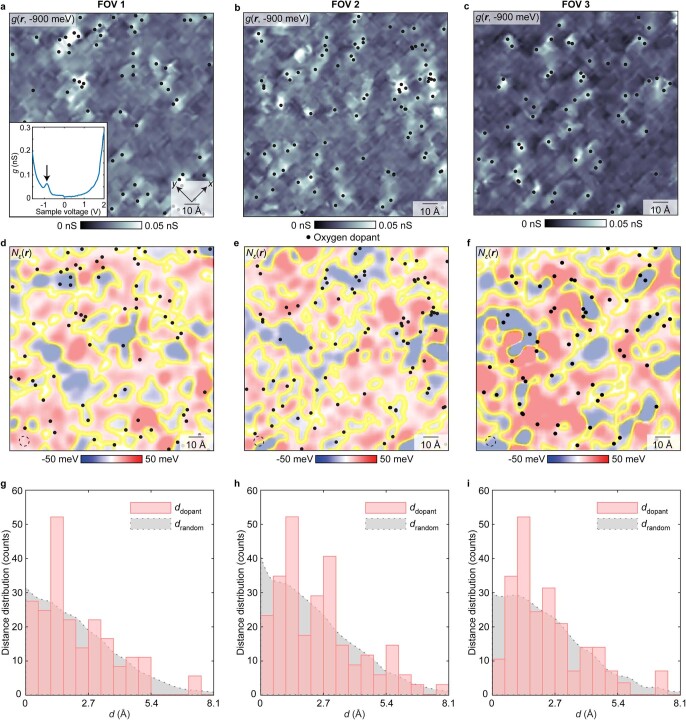
Statistical validation of dopant oxygen ion pinning of orbital ordered domains. (**a**–**c**) Three independent differential conductance maps $g\left({\boldsymbol{r}},-900{\rm{meV}}\right)$ where the locations of the oxygen dopants are identified as black circles. Inset of (a) shows a typical dI/dV spectrum of an oxygen dopant. (**d**–**f**) Oxygen-specific order parameter ${N}_{{\varepsilon }}\left({\boldsymbol{r}}\right)$ with overlaying oxygen dopants as black circles. The ${N}_{{\varepsilon }}\left({\boldsymbol{r}}\right)$ are measured simultaneously as $g$(***r***, -900 meV) in (**a**–**c**), where the domain walls are highlighted. (**g**–**i**) The ${d}_{{\rm{dopant}}}$ histogram (pink bar) is the distance from each dopant to the nearest location on the domain walls. The ${d}_{{\rm{random}}}$ histogram (grey curve) is the expectations of the distance between simulated random points and its nearest point in the domain walls.

Figure 3a shows our measured *N*_*ε*_(**r**) from the FOV shown in Fig. 2a, where a continuous image is achieved by the Gaussian smoothing of *N*_*ε*_(**R**_**i**__**j**_) with the radius shown by a small circle in the image. A strong breaking of intra-unit-cell *C*_4_ symmetry is now observed in δ*ε*(**r**), whereas the predominant disorder is revealed as Ising domains of opposite sign *N*_*ε*_ (a phenomenology that cannot be the result of anisotropy of the scan tip). The relative preponderance of the two orbitally ordered domains is about 2:1 in these *p* ≈ 0.17 hole-doped samples (Methods and Extended Data Figs. 3–5), indicating that the quenched disorder from dopant ions pins the orbitally ordered domains such that some domains of the opposite sign to the predominant order are stabilized. This intra-unit-cell structure in charge transfer energy implies a redistribution of electric charge that breaks rotational symmetry in the form of a charge quadrupole. But each such charge quadrupole has two possible orientations relative to the local environment, with two different energies (Fig. 3b): it is, thus, an electronic quadrupolar two-level system. Figure 3c shows the histogram of the measured *N*_*ε*_(**r**) in Fig. 3a, whose r.m.s. value is approximately 25 meV. The histogram of the measured |*N*_*ε*_| < 5 meV within the proposed Ising-domain walls is presented in Fig. 3d, representing a dense reservoir of thermally active quadrupolar two-level systems that should undergo rapid thermal fluctuations at finite temperatures. Such two-level systems occupy an area fraction of approximate 25% in this sample. Another issue is the size of the Ising domains, the largest of which (when averaged over the entire FOV; Methods and Extended Data Figs. 3–5) typically has a size of 30 nm^2^, equivalent to approximately 200 CuO_2_ unit cells. In the same FOV of these studies, we can identify the locations of interstitial oxygen dopant ions (Methods and Extended Data Fig. 7). When superimposed on the simultaneously measured *N*_*ε*_(**r**) images (Fig. 3e), we find a propensity for these ions to lie in the domain walls (Fig. 3f) between the orbitally ordered regions (Methods and Extended Data Fig. 7).

## Determination of energy splitting of O_*x*_ and O_*y*_ orbitals

**Fig. 4:**
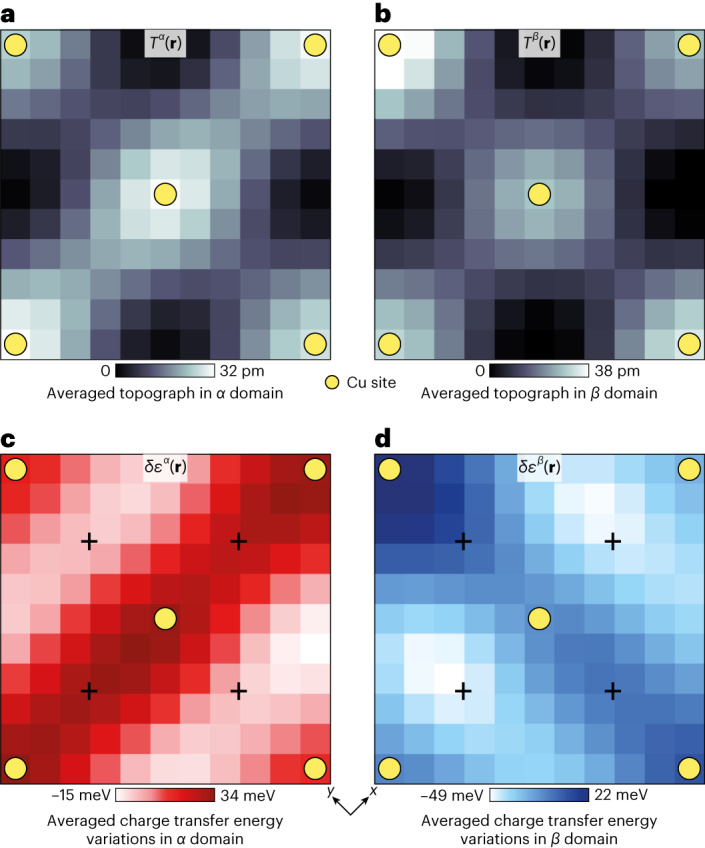
Sublattice-resolved charge transfer energy and orbital ordering. **a**, Unit-cell-averaged structure of *T*(**r**) averaged over all the regions shown in Fig. 3a where *N*_*ε*_ > +5 meV. **b**, Unit-cell-averaged structure of *T*(**r**) averaged over all the regions shown in Fig. 3a where *N*_*ε*_ < –5 meV. In both **a** and **b**, *C*_4_ symmetry is preserved. **c**, Unit-cell-averaged structure of δ*ε*(**r**) averaged over all the regions shown in Fig. 3a where *N*_*ε*_ > +5 meV. The charge transfer energy strongly breaks *C*_4_ rotation symmetry about every Cu site (yellow dots), and consequently, there is an energy splitting of approximately 50 meV between the charge transfer energies at the two crystal-equivalent oxygen sites (indicated by the crosses). Unprocessed high-voltage d*I*/d*V* images directly reveal the subtending data (Methods and Extended Data Fig. 6). **d**, Unit-cell-averaged structure of δ*ε*(**r**) averaged over all the regions shown in Fig. 3a where *N*_*ε*_ < –5 meV. Virtually identical phenomena as in **c**, but rotated by 90°. The difference between **c** and **d** is the difference in the internal structure of the CuO_2_ unit cell in the two distinct Ising domains of orbital order. Unprocessed high-voltage d*I*/d*V* images directly reveal the subtending data(Methods and Extended Data Fig. 6). Source data

**Extended Data Fig. 8:**
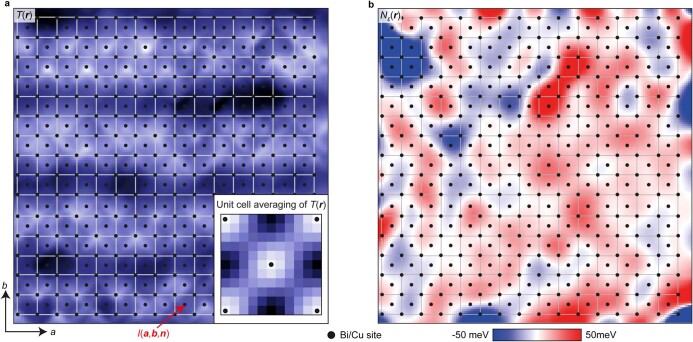
Unit-cell averaging (UCA) analysis. (**a**) Identification of the Cu site in the topograph $T\left({\boldsymbol{r}}\right)$. Each unit cell is defined by a Cu site (black circles) in the center and four Cu sites at the corners. The averaged image of all the unit cells is a UCA image presented in the inset. (**b**) The CuO_2_ unit cells are categorized into two zones of Ising domains from the parameter ${N}_{{\varepsilon }}\left({\boldsymbol{r}}\right)$ map. The UCA images of each Ising domain is subsequently calculated.

To illustrate the internal electronic structure of orbitally ordered CuO_2_ (Fig. 3a), we select all the regions with *N*_*ε*_ > +5 meV (domain *α*) and all the regions with *N*_*ε*_ < –5 meV (domain *β*). For each of these two zones, we first average the topographic image *T*(**r**) over all the CuO_2_ unit cells contained therein (Methods and Extended Data Fig. 8). Figure 4a,b shows that neither the topography nor the scan tip break the intra-unit-cell *C*_4_ symmetry. We then present δ*ε*(**r**) unit cell averaged over exactly the same two zones. The results (Fig. 4c,d) vividly demonstrate how strongly the intra-unit-cell rotational symmetry is broken at the charge transfer energy, within the two orthogonal Ising nematic domains. Focusing on the Cu sites (Fig. 4c,d) reveals the breaking of rotational symmetry, whereas the O_*x*_ and O_*y*_ sites (Fig. 4c,d) exhibit different charge transfer energies with separation on the 50 meV scale (Methods and Extended Data Figs. 3–5 report the repeatable observation). Indeed, this intra-unit-cell splitting of the charge transfer energies can be directly seen from the unprocessed data where the d*I*/d*V* spectra are separately identified at the O_*x*_ and O_*y*_ sites within the same unit cell (Methods and Extended Data Fig. 6). These results are indicative that the 2*p*^6^ oxygen orbital at O_*x*_ is separated from the upper Cu band by a different energy than that at O_*y*_, or equivalently, the existence of intra-unit-cell orbital order. Many characteristics of CuO_2_ intra-unit-cell orbital ordering become evident in Figs. 3 and 4: the spatial structure of δ*ε*(**r**) within CuO_2_ including the specific values at each Cu and inequivalent O site; the energy scale for these orbital-ordering phenomena near 50 meV; the statistical distribution δ*ε*(**r**) due to the short-correlation-length Ising domains; and the population of unit-cell-localized quadrupolar two-level systems whose energy barriers range continuously from zero. Overall, Figs. 3 and 4 show that the **Q** = 0 splitting between *ε*(**r**) at the two oxygen sites within a CuO_2_ unit cell is near 50 meV for Bi_2_Sr_2_CaCu_2_O_8+*x*_ samples with a hole density of *p* ≈ 0.17, but that long-range orbital order is absent resulting in two populations of Ising nematic domains.

These data may be compared with earlier X-ray scattering studies of orbital ordering across the family of La_2–*x*_Ba_*x*_CuO_4_ materials. In these crystals, signatures of intra-unit-cell symmetry breaking should occur as anisotropy in the scattering tensor at planar Cu sites. Here it is the intensity of the (001) scattering peak at the Cu L edge, which directly measures the electronic nematicity of the Cu 3*d* states within a single CuO_2_ plane and provides the indications of intra-unit-cell nematicity^31^. Figure 4c,d also shows that *C*_4_ symmetry is broken in the charge transfer electronic structure at the Cu sites (yellow dots), which—if due to Coulomb potentials inside the CuO_2_ unit cell—also indicates quadrupolar charge distributions surrounding the Cu sites, in agreement with other work^31^. With respect to other order parameters, we note that the orbitally ordered state at **Q** = 0 would not have any direct relation to the surface supermodulation or a perfectly harmonic charge density wave (CDW) state at finite **Q**_CDW_ = (2π/4*a*_0_, 0);(0, 2π/4*a*_0_) because any distinctions between O_*x*_ and O_*y*_ inside the unit cell are due to the density wave, average to 0 over one period^26,27^. However, higher harmonics of a CDW can, in principle, produce composite order parameters that break symmetry at **Q** = 0, as can ‘vestigial’ CDW states in the presence of disorder^32^. Another important subject is the effect of electron–lattice interactions: although the predominant theoretical motivation for such a state has been based on the repulsive *V*_*pp*_ potential between the oxygen atoms^2–9^ (Fig. 1b), it is inevitable that such orbital order will also couple to the B_1g_ phonon mode. If this mode softens in Bi_2_Sr_2_CaCu_2_O_8+*x*_, the O atom elevated above the CuO_2_ plane experiences a different potential than its neighbour, which is depressed below it^33^. One surprising new revelation is the distribution of spatially localized, low-activation-energy two-level systems within the boundaries between orbitally ordered domains. These are the electronic quadrupoles within each unit cell (Fig. 4c,d) whose energy barrier between the two orientations of *N*_*ε*_ tends to zero (Fig. 3d), making thermally activated fluctuations conceivable. Ultimately, as to the key issue of the 50 meV scale of energy splitting between O_*x*_:O_*y*_, its precise microscopic cause is an outstanding theoretical challenge apparently requiring realistically parameterized theory for the three-orbital model (equation (3)) within the CuO_2_ unit cell.

Nevertheless, important consequences do emerge from these studies. First, when physically realistic models of CuO_2_ electronic structure are used (for example, equation (1) with parameters relevant to real materials), Coulomb interactions between electrons at the two crystal-equivalent oxygen sites should generate intra-unit-cell rotational symmetry breaking, that is, orbital ordering^2–9^. By introducing sublattice-resolved *ε*(**r**) imaging techniques into CuO_2_ studies, this state has now been detected (Fig. 4). By visualizing an oxygen-site-specific nematic order parameter *N*_*ε*_(**r**), we reveal robust domains of *C*_2_ symmetry and with typical energy splitting of ~50 meV between the two intra-unit-cell oxygen sites (Fig. 3). Hence, a strong orbitally ordered state occurs in underdoped Bi_2_Sr_2_CaCu_2_O_8+*x*_ at the charge transfer energy scale. Spatially, the state is arranged in Ising domains that are highly disordered. This disorder appears to be bounded in shape and size by randomly sited oxygen dopant ions. Moreover, within the domain walls, as *N*_*ε*_→0 (Fig. 3d), we find an ensemble of low-energy-barrier electronic quadrupolar two-level systems. Most fundamentally, the microscopic mechanism proposed theoretically^2–9^ for the cuprate nematic phase, that is, orbital order between oxygen orbitals at the two separate oxygen sites of CuO_2_, is highly consistent with the observed intra-unit-cell rotational symmetry breaking of *ε*(**r**) that splits the energy between the two oxygen atoms by ~50 meV in Bi_2_Sr_2_CaCu_2_O_8+*x*._

## Methods

### Visualization of spatially modulating charge transfer energy

The spatially resolved charge transfer energy *ε*(**r**) may be visualized in cuprates using high-voltage^23,34,35^ differential tunnel conductance (*g*(**r**, *V*) ≡ d*I*/d*V*(**r**, *V*)) imaging of Bi_2_Sr_2_CaCu_2_O_8+*x*_ (ref. ^25^). Here *g*(**r**, *V*) was visualized in the range of –1.6 ≤ *V* ≤ 2.0 V and at a very high junction resistance of *R*_N_ ≈ 85 GΩ (*V*_S_ = –600 mV:*I*_S_ = 7 pA), which is necessary to suppress tip-induced electric-field effects. Figure 2b shows a typical example of a *g*(**r**, *V*) spectrum measured at *T* = 4.2 K (ref. ^25^). The edges of the filled lower band and the empty upper band can be identified from the appearance of an extremely rapid increase in the density of states. Here the value of charge transfer energy *ε*(**r**) is estimated at every location by subtracting these band edges at a constant differential conductance (*G* ≈ 20 pS) as follows:

4 $${\varepsilon }\left({\bf{r}}\right)={V}_{{\rm{empty}}}\left(G,{\bf{r}}\right)-{V}_{{\rm{filled}}}\left(G,{\bf{r}}\right).$$

Crystal supermodulation is a quasi-periodic lattice modulation along the (1, 1) direction in the crystal structure at wavevector **Q**_SM_. The resulting *ε*(**r**) map for Bi_2_Sr_2_CaCu_2_O_8+*x*_ shows strong modulations at the same wavevector, with an amplitude of ~0.3 eV, and has a spatial average value of 〈*ε*(**r**)〉 ≈ 1.2 eV, which agrees well with the charge transfer energy of Bi_2_Sr_2_CaCu_2_O_8+*x*_, as independently measured using a variety of different experimental techniques^24,36,37^. The analysis of the supermodulation influence on *ε*(**r**) and the corresponding supermodulation influence on the electron-pair density *n*_*p*_(**r**) measured by scanned Josephson tunnelling microscopy have revealed^25^ that the charge transfer energy *ε*(**r**) controls the density of condensed electron pairs *n*_*p*_(**r**) as ${\rm{d}}{\bar{n}}_{{{p}}}/{\rm{d}}{\varepsilon }$ ≈ –0.81 ± 0.17 eV^–1^.

Extended Data Fig. 1 compares the images of charge transfer energy *ε*(**r**) derived from the simple algorithm in equation (4) and the more general algorithm for charge transfer energy variation δ*ε*(**r**) used in the main text. The images of *ε* and δ*ε* bear high similarities in real space and reciprocal space. Their linecuts show identical and strong symmetry breaking at the Bragg peaks where the ratio of the intensity of the Bragg peak in the *y* direction to the Bragg peak in the *x* direction is $\frac{{{\bf{Q}}}_{y}}{{{\bf{Q}}}_{x}}=1.9$. Thus, our general algorithm for calculating the charge transfer energy variation δ*ε*(**r**) throughout this paper is consistent with the earlier simple algorithm^25^ for calculating *ε*(**r**). From Extended Data Fig. 1d,e, the δ*ε*(**r**) histogram features a narrower *σ* and therefore yields a higher signal-to-noise ratio. Hence, we implement our algorithm to measure the charge transfer energy variation δ*ε*(**r**) throughout.

### Experimental evidence of Q = 0 rotational symmetry breaking in cuprates

The existence of a nematic state in cuprates has been extensively demonstrated on the basis of multiple experimental techniques (Fig. 1a). Neutron scattering, particularly with polarized neutrons, is extensively used to detect anisotropy at Bragg peaks, indicating intra-unit-cell (IUC) symmetry breaking^38–43^. Angle-resolved photoemission spectroscopy^44–46^ shows that above T*, left- and right-circularly polarized light have the same intensity at the mirror plane, but when cooled below T*, a difference in intensities of the left- and right-circularly polarized light at the mirror plane emerges, which is a signature of **Q** = 0 rotational symmetry breaking. Torque magnetometry studies^47,48^ have reported a kink of the in-plane anisotropy of susceptibility at T*, which also relates to rotational symmetry breaking. Optical anisotropy^49^ measurements show a substantial change in the second harmonic of the optical response near T*, whereas the linear response remains unchanged in the same region. This could be explained if bulk inversion symmetry was broken; alongside this, nematicity is also seen. In polarization-resolved Raman scattering^50^, the suppression of susceptibility near T* is observed. Elastoresistance measurements^51^ of in-plane anisotropy that onsets near T* also indicate a nematic state. Resonant ultrasound spectroscopy^52^ finds a phase transition at T* by noting discontinuities in the frequencies and widths of the vibrational normal modes of a crystal. Scanning tunnelling microscopy experiments^53^ have detected the intra-unit-cell rotational symmetry breaking in the low-energy density of states, and this nematicity exhibits Ising domains that diminish in size and intensity approaching the pseudogap endpoint. In La_1.875_Ba_0.125_CuO_4_ (refs. ^31,54^), La_1.65_Eu_0.2_Sr_0.15_CuO_4_ (refs. ^31,55^) and La_1.6–*x*_Nd_0.4_Sr_*x*_CuO_4_ (refs. ^31,55,56^), resonant soft-X-ray scattering also reveals a nematic phase. This electronic phase transition is explored using resonant X-ray scattering, particularly measuring the [001] Bragg peak. The intensity of the Bragg peak is proportional to the electronic nematic order parameter that is associated with the symmetry breaking at the Cu *d* state. When the photon energies sensitive to Cu are used, the emerging [001] intensity reveals a nematic phase. Importantly, a nematic phase is reported across numerous cuprate families, including YBa_2_Cu_3_O_7–*x*_ (refs. ^38,39,42,43,47,49,52^), Bi_2_Sr_2_CaCu_2_O_8+*x*_ (refs. ^44,46,50,51,53^), Bi_2_Sr_2_CuO_6+*x*_ (ref. ^45^), Bi_2–*z*_Pb_*z*_Sr_2–*y*_La_*y*_CuO_6+*x*_ (ref. ^57^), HgBa_2_CuO_4+*δ*_ (refs. ^40,41,48^) and La_1.875_Ba_0.125_CuO_4_ (refs. ^31,54,56^). But its microscopic mechanism was unknown.

### Visualization of charge transfer energy variations

The averaged differential conductance *g*(*V*) is shown as a dashed curve in Extended Data Fig. 1f,g. The variations in charge transfer energy are determined from the deviation of a point spectrum from the averaged spectrum. The states on the *V* > 0 side have a positive integral ${I}_{+}\left({\bf{r}}\right)={\int }_{0}^{{V}_{\max }}g(V,{\bf{r}}){{\rm{d}}V}$, where the maximum energy is *V*_max_ = 2 V. The states on the *V* < 0 side have a negative integral ${I}_{-}\left({\bf{r}}\right)={\int }_{{V}_{\min }}^{0}g(V,{\bf{r}})$ where the minimum energy is *V*_min_ = –1.6 V. The variation in each integral *I*_+_(**r**) and *I*_–_(**r**) from the average values $\bar{{I}_{+}}$ and $\bar{{I}_{\pm }}$, respectively, occurs due to the variation in energy separation δ*ε*(**r**) between the lower and upper bands from its average value. To efficiently evaluate the energy splitting between the point spectrum and averaged spectrum, the integral difference is normalized by the difference between the maximum differential conductance *g*_max_ and the minimum difference conductance *g*_min_. Here *g*_max_ = 0.220 nS is given by the maximum of the FOV-averaged spectrum and *g*_min_ = 0.009 nS is the minimum differential conductance of the averaged spectrum.

Hence, we define

5 $$(\;{g}_{\max }-{g}_{\min })\delta {\rm{\varepsilon }}\left({\bf{r}}\right)=\left[\bar{{I}_{+}}-{I}_{+}\left({\bf{r}}\right)\right]-[\bar{{I}_{-}}-{I}_{-}\left({\bf{r}}\right)].$$

There are the two typical cases shown in Extended Data Fig. 1f,g.

### LF symmetrization of charge transfer energy images

To correct picometre distortions due to piezo-electronic drift in scanning, we apply the affine transformations of the LF algorithm^26^. This is the key for visualizing the intra-unit-cell electronic structure. To obtain this goal, the dataset with atomic resolution and the dataset with high voltage (–1.6 to 2.0 V) must be registered with atomic accuracy. The two datasets are measured in the same FOV. The topographs of the two datasets, namely, *T*_1_′(**r**) in the atomic-resolution dataset (Extended Data Fig. 2a) and *T*_2_′(**r**) in the high-voltage dataset (Extended Data Fig. 2b), are used to register identical atoms in one dataset with those in the other. LF is applied to produce the corrected images of both topographs. Subsequently, both topographs are registered by spatial translations, whose accuracy is evaluated by their cross-correlation image. *T*_1_(**r**) in Extended Data Fig. 2e and *T*_2_(**r**) in Extended Data Fig. 2f are now registered. All the transformation parameters applied to *T*_2_′(**r**) are subsequently applied to the high-voltage differential conductance map *g*′(**r**, *V*) that is simultaneously measured with the topography. The differential conductance map *g*(**r**, *V*) is registered.

Extended Data Fig. 2a,b shows two topographs of a Bi_2_Sr_2_CaCu_2_O_8+*x*_ surface taken with atomic resolution and high voltage, respectively. They are LF corrected and registered with atomic resolution (Fig. 2e,f). The precision of the image registration is shown in Extended Data Fig. 2f (inset). The maximum of the cross-correlation between *T*_1_(**r**) and *T*_2_(**r**) coincides with the (0, 0) cross-correlation vector (inset of Extended Data Fig. 2f). The offset of the two registered images are within three pixels, meaning that the precision of registration is better than 80 pm everywhere in the whole FOV.

To demonstrate that the evidence of rotational symmetry breaking is present in the unprocessed charge transfer energy variation δ*ε*′(**r**), the unprocessed data of δ*ε*′(**r**) (Extended Data Fig. 2c,d) and processed δ*ε*(**r**) (Extended Data Fig. 2g,h) are compared. δ*ε*′(**r**) is calculated from the unprocessed dataset and δ*ε*(**r**) is calculated from the LF-corrected and registered dataset. The Fourier transform δ*ε*′(**q**) of the unprocessed data shows anisotropy at the Bragg peaks. The processed data δ*ε*(**q**) shows the same anisotropic Bragg peaks and the background noise is much lower in the drift-corrected data than in the unprocessed data. Thus, the LF algorithm does not alter the conclusion that *C*_4_ symmetry is broken in δ*ε*.

### Intra-unit-cell charge transfer energy imaging: repeatability and reliability

Here we first compare the result of multiple experiments to evaluate the repeatability of the phenomena reported. First, the Fourier analysis was repeated on three different FOVs of the same sample (Extended Data Figs. 3–5). The topograph of each FOV is shown in Extended Data Fig. 3a–5a. The *T*(**r**) value is an atomically resolved topograph that is registered with the differential conductance map *g*(**r**, *V*). The PSD Fourier transform of the topograph is then shown in Extended Data Figs. 3b–5b. The Bragg peaks **Q**_*x*_ and **Q**_*y*_ are of similar magnitude in the topograph; therefore, *C*_4_ symmetry is preserved, indicating that neither the lattice nor the scanning tip produces IUC symmetry breaking. The red linecut of *T*(**q**) is taken from **q** = (0, 0) to (1, 0)2π/*a*_0_ and the blue linecut is taken from **q** = (0, 0) to (0, 1)2π/*a*_0_ (Extended Data Figs. 3c–5c). These linecuts quantitatively and clearly show that **Q**_*x*_ ≈ **Q**_*y*_ in *T*(**q**) of all the FOVs. This confirms that the tip and lattice preserve *C*_4_ symmetry.

Extended Data Figs. 3d–5d show the charge transfer energy variations δ*ε*(**r**). All the FOVs show high levels of disorder, and the same energy ranges from –300 to +200 meV. Next, δ*ε*(**q**), the PSD Fourier transform of charge transfer energy variation, is shown in Extended Data Figs. 3e–5e. In all the FOVs, it is clear that the Bragg peaks at (0, 1)2π/*a*_0_ are more intense than the peaks at (1, 0)2π/*a*_0_, meaning that IUC *C*_4_ rotational symmetry is broken. Linecuts of δ*ε*(**q**), taken from **q** = (0, 0) to the Bragg peaks, show clear anisotropy between the intensities of Bragg peaks in all the FOVs (Extended Data Figs. 3f–5f). Therefore, IUC rotational symmetry is broken in δ*ε*. The histograms of δ*ε* are asymmetric and the peak of the histogram is shifted from 0 in all the FOVs (Extended Data Figs. 3f–5f, inset), which is consistent with the anisotropy in charge transfer energy variations δ*ε*.

Collectively, the anisotropy in Bragg peaks of the charge transfer energy variations δ*ε* is repeatable in multiple experiments at the same hole density. The δ*ε* values from independent FOVs show similar statistics. The nematicity is not generated by the crystallography or the scanning tip. Therefore, the Fourier analysis of δ*ε* appears robust and reliable.

Second, the oxygen-site-specific imaging *N*_*ε*_(**r**) of the charge transfer are repeated for the three FOVs shown in Extended Data Figs. 3g–5g. This focuses attention on the charge transfer symmetry breaking occurring on the planar oxygen sites. The three FOVs show disordered Ising domains. The relative strength between the two domains is defined as the area ratio *A*_red_/*A*_blue_, which is approximately 2.1 ± 0.2. This is consistent with the relative strength between the two Bragg peaks in the PSD Fourier transform of δ*ε*, that is, $\frac{\delta {\varepsilon }\left({{\bf{Q}}}_{y}\right)}{\delta {\varepsilon }\left({{\bf{Q}}}_{x}\right)}$ ≈ 1.9 ± 0.3 in Extended Data Figs. 3f–5f. This result demonstrates that the relative preponderance of the two orbitally ordered domains is approximately 2:1 in the 17% hole-doped sample. The histograms of *N*_*ε*_(**r**) in the inset of these figures are asymmetric and the peak of the histogram is shifted from *N*_*ε*_ = 0. This observation is consistent with the data in Extended Data Figs. 3g–5g that the preponderance of the *N*_*ε*_(**r**) > 0 domain is stronger than the *N*_*ε*_(**r**) < 0 domain. The r.m.s. value of the IUC energy splitting between the O_*x*_ and O_*y*_ sites ranges from 20 to 30 meV.

The microscopic structure inside the Ising domains is visualized from the unit-cell-averaged δ*ε*(**r**) images of each domain (Extended Data Figs. 3h,i–5h,i). The unit-cell-averaged δ*ε*^*α*^(**r**) from the *N*_*ε*_(**r**) > 5 meV region is indicated in Extended Data Figs. 3h–5h, and the unit-cell-averaged δ*ε*^*β*^(**r**) from the *N*_*ε*_(**r**) < –5 meV is indicated in Extended Data Figs. 3i–5i. Clearly, the charge transfer energy variations break the IUC rotational symmetry. Because the number of unit cells in the *N*_*ε*_(**r**) > 5 meV domain is almost twice that in the other domain, the signal-to-noise ratio is higher in unit-cell averaging than that in $\delta {{\varepsilon }}_{B}^{R}({\bf{r}})$. The unit-cell-averaged images of the topograph *T*(**r**) serve (Extended Data Figs. 3h,i–5h,i (insets)) as a reference of δ*ε*^*α*^(**r**) and δ*ε*^*β*^(**r**). The tip preserves IUC rotational symmetry and the crystallography of the CuO_2_ unit cell does not break rotational symmetry.

Third, from the unprocessed data (Extended Data Fig. 6), we directly demonstrate that the rotational symmetry breaking reported here is a basic property of the charge transfer gap. Three representative examples of this from three different but perfectly typical FOVs are presented in Extended Data Fig. 6. These same phenomena are omnipresent throughout the FOVs in the experiment, meaning that orbital ordering is universal.

We show the topography, the orbital order parameter *N*_*ε*_(**r**) and 20 unprocessed d*I*/d*V* point spectra from each FOV. The first column in Extended Data Fig. 6 is the topographs showing the FOVs where the charge transfer measurements are taken. The locations of five CuO_2_ unit cells consisting of ten different oxygen sites are highlighted in this figure (blue and red dots). The second column in Extended Data Fig. 6 shows the nematic domains *N*_*ε*_(**r**) in this FOV. The third column in Extended Data Fig. 6 shows domain 1 (red), where the O_*x*_ spectra are shifted in the range from almost −50 to −30 meV with respect to the O_*y*_ spectra inside the same unit cell. The charge transfer gap on the O_*x*_ sites (red) is higher than the gap on the O_*y*_ site (blue) in this domain. Domain 2 (blue) shows that the O_*y*_ spectra are shifted in the range from almost −50 to −30 meV with respect to the O_*x*_ spectra (Extended Data Fig. 6, fourth column), which means that the O_*y*_ sites have higher charge transfer energy than the O_*x*_ sites in this domain. The two domains are separated by a domain wall. Such domains are observed throughout the unprocessed data, showing the intra-unit-cell symmetry breaking and Ising domains of orbital ordering.

### Dopant-oxygen-ion pinning of orbitally ordered domains

To explore the causes for obvious heterogeneity in the orbitally ordered domains, we search for the dopant oxygen ions and their relationship with the domains. Each dopant oxygen ion is identified as a maximum located at –900 meV in the differential conductance spectrum (Extended Data Fig. 7a, inset). A total of ~70 oxygen dopants are found in the differential conductance map *g*(**r**, –900 meV) with an image size of 17 × 17 nm^2^ (Extended Data Fig. 7a). The locations of the oxygen dopant are overlaid onto the simultaneously measured IUC oxygen-specific nematic order parameter *N*_*ε*_(**r**) (Extended Data Fig. 7d); visually, we see that the oxygen dopants are near the *N*_*ε*_ domain walls (yellow contours).

The distance *d*_dopant_ between each oxygen dopant and their nearest location on the domain walls are calculated and a distribution is created (Extended Data Fig. 7g). To validate that the oxygen dopants are located near the *N*_*ε*_ domain walls, the distribution of *d*_dopant_ is compared with the distance *d*_random_ between randomly generated points and the domain walls. The random points are generated from a two-dimensional Poisson-disc sampling function and the random points are separated from each other. The random points have no spatial correlation with the orbitally ordered domains. The expected averaged distance *d*_random_ to the domain walls is calculated and compared with *d*_dopant_ (Extended Data Fig. 7g).

The distribution of *d*_dopant_ is different from *d*_random_ with regard to two aspects. Although the *d*_dopant_ distribution has a sharp peak at 1.6 Å, the *d*_random_ distribution has a blunt plateau. The deviation in the distance distribution clearly indicates that the oxygen dopants are located near the domain walls of the orbitally ordered domains, providing statistical evidence that they are pinning the nematic domains.

We repeated the above statistical analysis for three independent FOVs (Extended Data Fig. 7a–c). The data in Extended Data Fig. 7f are also presented in Fig. 3e. The sum of the three histograms (Extended Data Fig. 7g–i) is presented in Fig. 3f. A total of 237 oxygen dopants are studied in the total histogram.

### Visualizing intra-unit-cell charge transfer order parameter

The unit cells inside one Ising domain are averaged to increase the signal-to-noise ratio of the microstructure of the IUC symmetry breaking order^58^. First, each CuO_2_ unit cell is identified in the topography *T*(**r**) (Extended Data Fig. 8a, grey grid). Each unit cell has a size of 10 × 10 pixels^2^ and is labelled as *I*(**a**, **b**, **n**). The image series *I*(**a**, **b**, **n**) is categorized into two datasets, namely, *I*_1_(**a**, **b**, **n**) and *I*_2_(**a**, **b**, **n**). Here *I*_1_ is inside a positive domain with *N*_*ε*_(**r**) > 5 meV and *I*_2_ is inside a negative domain with *N*_*ε*_(**r**) < –5 meV (Extended Data Fig. 8b). The images *I*_1,2_(**a**, **b**, **n**) have lateral dimensions *x* and *y* and a counting index *n*. *T*(**r**) has already been atomically registered and the lattice displacements have been corrected using the LF algorithm, making each lattice square and periodic. The image set of *I*_1,2_(**a**, **b**, **n**) comprises *n* images of *I*_1,2_(**a**, **b**). The images are independently cropped from *T*(**r**), where each image has a size of 11 × 11 pixels^2^ (5.9 × 5.9 Å^2^) and corresponds to one CuO_2_ unit cell rotated by 45°. The single CuO_2_ unit cell is defined by the location of Bi/Cu atoms (Extended Data Fig. 8).

### Orbitally ordered domains are unaffected by supermodulation

**Extended Data Fig. 9:**
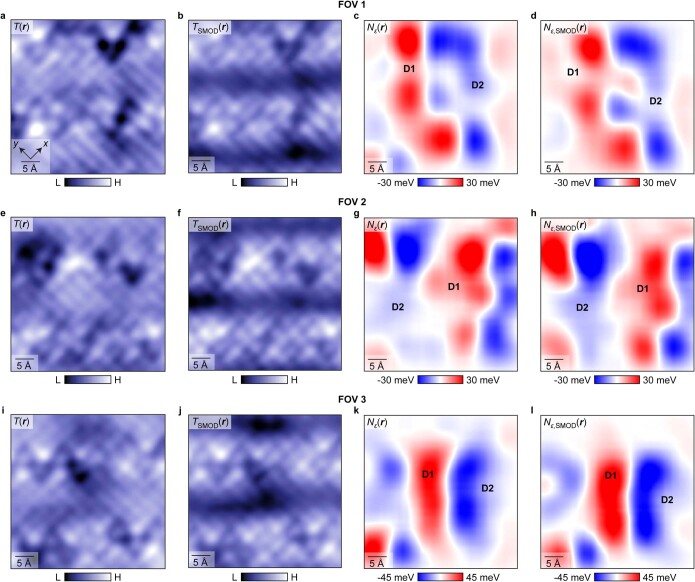
Supermodulation does not affect orbital ordering domains. **(a, e, i)** show the topographs where the supermodulation is filtered. **(b, f, j)** present the topographs where the supermodulation is kept. **(c, g, k)** show the orbital ordering domains calculated from the topographs in the first column where the supermodulation is removed. **(d, h, l)** show the orbital ordering domains calculated from the topographs in the second column where the supermodulation remains. The orbital ordering domains from the third (without supermodulation) and fourth columns (with supermodulation) are virtually identical. The supermodulation has virtually no effect on the orbital-order parameter used in this paper.

To further illustrate the lack of effect that supermodulation has on **Q** = 0 orbital ordering, we have compared the topographs and orbitally ordered domains obtained with and without supermodulation from three independent FOVs (Extended Data Fig. 9). The first column in Extended Data Fig. 9 shows the topographs where the supermodulation is removed via Fourier filtering at the **Q**_SM_ peak. Their orbitally ordered domains are presented in the third column (Extended Data Fig. 9). The second column in Extended Data Fig. 9 presents the topographs where supermodulation is retained in the analysis. Although the atomic structure is strongly modulated by supermodulation, the orbitally ordered domains in the fourth column (Extended Data Fig. 9) remain virtually the same as the orbitally ordered domains where the **Q**_SM_ peak is filtered, as shown in the third column.

## Online content

Any methods, additional references, Nature Portfolio reporting summaries, source data, extended data, supplementary information, acknowledgements, peer review information; details of author contributions and competing interests; and statements of data and code availability are available at 10.1038/s41563-024-01817-z.

### Source data

Source Data Fig. 1 Analysed data used to plot Fig. 1a.

Source Data Fig. 2 Analysed data used to plot Fig. 2a–h.

Source Data Fig. 3 Analysed data used to plot Fig. 3a,c–f.

Source Data Fig. 4 Analysed data used to plot in Fig. 4a–d.

## Data Availability

The data shown in the main figures are available via Zenodo at 10.5281/zenodo.10510971. Source data are provided with this paper.

## Code availability

The code is available via Zenodo at 10.5281/zenodo.10515736.

## References

[1] Fradkin E, Kivelson SA, Tranquada JM (2015). Colloquium: theory of intertwined orders in high temperature superconductors. Rev. Mod. Phys..

[2] Kivelson SA, Fradkin E, Geballe TH (2004). Quasi-one-dimensional dynamics and nematic phases in the two-dimensional Emery model. Phys. Rev. B.

[3] Fischer MH, Kim EA (2011). Mean-field analysis of intra-unit-cell order in the Emery model of the CuO_2_ plane. Phys. Rev. B.

[4] Bulut S, Atkinson WA, Kampf AP (2013). Spatially modulated electronic nematicity in the three-band model of cuprate superconductors. Phys. Rev. B.

[5] Fischer MH, Wu S, Lawler M, Paramekanti A, Kim EA (2014). Nematic and spin-charge orders driven by hole-doping a charge-transfer insulator. New J. Phys..

[6] Maier TA, Scalapino DJ (2014). Pairing interaction near a nematic quantum critical point of a three-band CuO_2_ model. Phys. Rev. B.

[7] Tsuchiizu M, Kawaguchi K, Yamakawa Y, Kontani H (2018). Multistage electronic nematic transitions in cuprate superconductors: a functional-renormalization-group analysis. Phys. Rev. B.

[8] Chiciak A, Vitali E, Shi H, Zhang S (2018). Magnetic orders in the hole-doped three-band Hubbard model: spin spirals, nematicity, and ferromagnetic domain walls. Phys. Rev. B.

[9] Yamase H (2021). Theoretical insights into electronic nematic order, bond-charge orders, and plasmons in cuprate superconductors. J. Phys. Soc. Jpn.

[10] Krüger F, Kumar S, Zaanen J, Van Den Brink J (2009). Spin-orbital frustrations and anomalous metallic state in iron-pnictide superconductors. Phys. Rev. B.

[11] Lv W, Wu J, Phillips P (2009). Orbital ordering induces structural phase transition and the resistivity anomaly in iron pnictides. Phys. Rev. B.

[12] Lee CC, Yin WG, Ku W (2009). Ferro-orbital order and strong magnetic anisotropy in the parent compounds of iron-pnictide superconductors. Phys. Rev. Lett..

[13] Fernandes RM, Chubukov AV, Schmalian J (2014). What drives nematic order in iron-based superconductors?. Nat. Phys..

[14] Hirschfeld PJ, Korshunov MM, Mazin II (2011). Gap symmetry and structure of Fe-based superconductors. Rep. Prog. Phys..

[15] Sprau PO (2017). Discovery of orbital-selective Cooper pairing in FeSe. Science.

[16] Yi M, Zhang Y, Shen ZX, Lu D (2017). Role of the orbital degree of freedom in iron-based superconductors. npj Quantum Mater..

[17] Fernandes RM (2022). Iron pnictides and chalcogenides: a new paradigm for superconductivity. Nature.

[18] Emery, V. J. Theory of high-*T*_c_ superconductivity in oxides. *Phys. Rev. Lett.* **58**, 2794–2797 (1987). 10.1103/PhysRevLett.58.2794 10034851

[19] Littlewood PB, Varma CM, Abrahams E (1989). Pairing instabilities of the extended Hubbard model for Cu-O based superconductors. Phys. Rev. Lett..

[20] Tallon, J. L. & Loram, J. W. The doping dependence of *T**—what is the real high-*T*_c_ phase diagram? *Physica C* **349**, 53–68 (2001).

[21] Norman MR, Pines D, Kallin C (2005). The pseudogap: friend or foe of high *T*_c_?. Adv. Phys..

[22] Fradkin E, Kivelson SA, Lawler MJ, Eisenstein JP, Mackenzie AP (2010). Nematic Fermi fluids in condensed matter physics. Annu. Rev. Condens. Matter Phys..

[23] Cai P (2016). Visualizing the evolution from the Mott insulator to a charge-ordered insulator in lightly doped cuprates. Nat. Phys..

[24] Ruan W (2016). Relationship between the parent charge transfer gap and maximum transition temperature in cuprates. Sci. Bull..

[25] Mahony SMO (2022). On the electron pairing mechanism of copper-oxide high temperature superconductivity. Proc. Natl Acad. Sci. USA.

[26] Lawler MJ (2010). Intra-unit-cell electronic nematicity of the high-*T*_c_ copper-oxide pseudogap states. Nature.

[27] Fujita K (2014). Direct phase-sensitive identification of a *d*-form factor density wave in underdoped cuprates. Proc. Natl Acad. Sci. USA.

[28] Hamidian MH (2016). Atomic-scale electronic structure of the cuprate *d*-symmetry form factor density wave state. Nat. Phys..

[29] Mesaros A (2011). Topological defects coupling smectic modulations to intra-unit-cell nematicity in cuprates. Science.

[30] Mukhopadhyay S, Sharma R, Koo C, Edkins SD, Hamidian MH (2019). Evidence for a vestigial nematic state in the cuprate pseudogap phase. Proc. Natl Acad. Sci. USA.

[31] Achkar AJ (2016). Nematicity in stripe-ordered cuprates probed via resonant X-ray scattering. Science.

[32] Nie L, Tarjus G, Kivelson SA (2014). Quenched disorder and vestigial nematicity in the pseudogap regime of the cuprates. Proc. Natl Acad. Sci. USA.

[33] Banerjee S, Atkinson WA, Kampf AP (2020). Emergent charge order from correlated electron-phonon physics in cuprates. Commun. Phys..

[34] McElroy K (2005). Atomic-scale sources and mechanism of nanoscale electronic disorder in Bi_2_Sr_2_CaCu_2_O_8+*δ*_. Science.

[35] Kohsaka Y (2012). Visualization of the emergence of the pseudogap state and the evolution to superconductivity in a lightly hole-doped Mott insulator. Nat. Phys..

[36] Yang S (2017). Revealing the Coulomb interaction strength in a cuprate superconductor. Phys. Rev. B.

[37] Itoh T, Fueki K, Tanaka Y, Lhara H (1999). Optical conductivity spectra and electronic structure of Bi_2_Sr_2_(Y_1–*x*_Ca_*x*_)Cu_2_O_*y*_ system. J. Phys. Chem. Solids.

[38] Fauqué B (2006). Magnetic order in the pseudogap phase of high-*T*_c_ superconductors. Phys. Rev. Lett..

[39] Mook HA, Sidis Y, Fauqué B, Balédent V, Bourges P (2008). Observation of magnetic order in a superconducting YBa_2_Cu_3_O_6.6_ single crystal using polarized neutron scattering. Phys. Rev. B.

[40] Li Y (2008). Unusual magnetic order in the pseudogap region of the superconductor HgBa_2_CuO_4+*δ*_. Nature.

[41] Li Y (2011). Magnetic order in the pseudogap phase of HgBa_2_CuO_4+*δ*_ studied by spin-polarized neutron diffraction. Phys. Rev. B.

[42] Mangin-Thro L, Sidis Y, Wildes A, Bourges P (2015). Intra-unit-cell magnetic correlations near optimal doping in YBa_2_Cu_3_O_6.85_. Nat. Commun..

[43] Mangin-Thro L, Li Y, Sidis Y, Bourges P (2017). *a-b* anisotropy of the intra-unit-cell magnetic order in YBa_2_Cu_3_O_6.6_. Phys. Rev. Lett..

[44] Kaminski A (2002). Spontaneous breaking of time-reversal symmetry in the pseudogap state of a high-*T*_c_ superconductor. Nature.

[45] He RH (2011). From a single-band metal to a high-temperature superconductor via two thermal phase transitions. Science.

[46] Nakata S (2021). Nematicity in a cuprate superconductor revealed by angle-resolved photoemission spectroscopy under uniaxial strain. npj Quantum Mater..

[47] Sato Y (2017). Thermodynamic evidence for a nematic phase transition at the onset of the pseudogap in YBa_2_Cu_3_O_*y*_. Nat. Phys..

[48] Murayama H (2019). Diagonal nematicity in the pseudogap phase of HgBa_2_CuO_4+*δ*_. Nat. Commun..

[49] Zhao L (2017). A global inversion-symmetry-broken phase inside the pseudogap region of YBa_2_Cu_3_O_*y*_. Nat. Phys..

[50] Auvray N (2019). Nematic fluctuations in the cuprate superconductor Bi_2_Sr_2_CaCu_2_O_8+*δ*_. Nat. Commun..

[51] Ishida K (2020). Divergent nematic susceptibility near the pseudogap critical point in a cuprate superconductor. J. Phys. Soc. Jpn.

[52] Shekhter A (2013). Bounding the pseudogap with a line of phase transitions in YBa_2_Cu_3_O_6+*δ*_. Nature.

[53] Fujita K (2014). Simultaneous transitions in cuprate momentum-space topology and electronic symmetry breaking. Science.

[54] Achkar AJ (2016). Orbital symmetry of charge-density-wave order in La_1.875_ Ba_0.125_CuO_4_ and YBa_2_Cu_3_O_6.67_. Nat. Mater..

[55] Gupta NK (2021). Vanishing nematic order beyond the pseudogap phase in overdoped cuprate superconductors. Proc. Natl Acad. Sci. USA.

[56] Achkar AJ (2013). Resonant X-ray scattering measurements of a spatial modulation of the Cu 3*d* and O 2*p* energies in stripe-ordered cuprate superconductors. Phys. Rev. Lett..

[57] Song C-L (2023). Critical nematic correlations throughout the doping range in Bi_2−*z*_Pb_*z*_Sr_2−*y*_La_*y*_CuO_6+*x*_. Nat. Commun..

[58] Jones, L., Wang, S., Hu, X., ur Rahman, S. & Castell, M. R. Maximising the resolving power of the scanning tunneling microscope. *Adv. Struct. Chem. Imaging* **4**, 7 (2018). 10.1186/s40679-018-0056-7 PMC5992247 29930895

